# Life-course body shape trajectories and cerebral oxygen metabolism in community-dwelling older adults

**DOI:** 10.1007/s11357-025-02082-x

**Published:** 2026-01-12

**Authors:** Yifan Yan, Yaping Zhang, Xuhao Zhao, Renwei Chen, Shenghao Fang, Yi Zhou, Jingkai Huang, Fuyan Wang, Christopher Chen, Zixuan Lin, Xin Xu

**Affiliations:** 1 School of Public Health, The Second Affiliated Hospital of School of Medicine, Zhejiang University, No. 866, Yuhangtang Road, Hangzhou, 310058 Zhejiang China; 2 Nanhu Brain-computer Interface institute, Hangzhou, Zhejiang China; 3 Department of Radiology, Sir Run Run Shaw Hospital, Zhejiang University School of Medicine, Hangzhou, Zhejiang China; 4 Memory, Ageing, and Cognition Centre (MACC), Department of Pharmacology, Yong Loo Lin School of Medicine, National University of Singapore, Singapore, Singapore; 5 Key Laboratory for Biomedical Engineering of Ministry of Education, Department of Biomedical Engineering, College of Biomedical Engineering & Instrument Science, Zhejiang University, Hangzhou, Zhejiang China; 6 Key Laboratory of Intelligent Preventive Medicine of Zhejiang Province, Hangzhou, China; 7 The State Key Lab of Brain-Machine Intelligence, MOE Frontier Science Center for Brain Science and Brain-Machine Integration, Zhejiang University School of Medicine, Hangzhou, China

**Keywords:** Adiposity, Cerebral metabolic rate of oxygen, Oxidative stress, Hypothalamus, Group-based trajectories

## Abstract

Obesity and lifelong body-shape fluctuation are associated with late-life structural brain damage, suggesting the involvement of metabolic pathways. The cerebral metabolic rate of oxygen (CMRO₂) reflects hemodynamic and oxidative stress and precedes structural atrophy, but its role in adiposity-related brain change remains unclear. We examined whether current and life-course adiposity relate to CMRO₂ and to structural change. A total of 303 community-dwelling adults aged 50 years and older were included. Body shape was assessed using Body Mass Index (BMI) and Body Roundness Index (BRI). Global CMRO₂ was derived from TRUST and phase-contrast MRI. T1-weighted MPRAGE provided volumetry, and medial temporal atrophy (MTA) grading. General linear models estimated associations of BMI and BRI with CMRO₂, including age interactions. Age-stratified mediation tested CMRO₂ as a mediator of adiposity to MTA associations. Body-shape trajectories at ages 25, 40, 60, and current age were modeled and related to CMRO₂ and metabolism-related regions. Adiposity was associated with lower CMRO₂: with overweight (β = -1.12 μmol/100 g/min, 95%CI = (-1.96, -0.28)) and higher BRI (β = -1.31, 95%CI = (-2.36, -0.27)) showing stronger effects with advancing age. Among participants aged 70 years, CMRO₂ mediated the association between BMI and MTA (indirect β = 0.06, 95%CI = (0.01, 0.14)). Three adulthood body-shape patterns emerged, and CMRO₂ was lower in moderate increasing (β = -11.40; 95%CI = (-20.90, -1.90)) and high-rising (β =  − 12.23; 95%CI = (-23.56, -0.90)) groups. Metabolism-related regions were larger in higher-risk patterns, particularly the left hypothalamus. Greater and prolonged adiposity is linked to reduced CMRO₂ and related structural differences in older adults.

**Supplementary information:**

The online version contains supplementary material available at 10.1007/s11357-025-02082-x.

## Introduction

Later-life obesity, particularly in the abdominal region, has been associated with structural imaging markers of neurodegeneration, such as atrophy and white matter burden [1–5]. Before overt structural damage occurs, cerebral metabolic rate of oxygen (CMRO₂) serves as an index of cerebral oxygen homeostasis and has been increasingly recognized as a sensitive biomarker of brain aging and neurodegeneration [6–8]. Importantly, CMRO₂ can be measured noninvasively with MRI in a relatively short period of time and without the need for contrast agents or radioactive tracers [7, 9], providing global information on cerebral oxygen metabolism and enabling population-level assessment. In older adults, obesity is linked to reductions in cerebral perfusion [10] and to low-grade, inflammation-related oxidative stress [11]. These alterations indicate microvascular dysfunction and impaired neurovascular coupling, which reduce oxygen-delivery efficiency and impose a cerebral metabolic burden that may accelerate neurodegenerative processes [12]. In this context, CMRO₂ offers a modifiable and monitorable biomarker for earlier risk stratification and for evaluating brain metabolic dysfunction before irreversible structural damage accrues. Yet whether and how obesity relates to CMRO₂ in the aging population remains poorly characterized.

Beyond later life, obesity during midlife and even early adulthood has also been associated with adverse brain outcomes, including smaller brain volumes [13] and accelerated brain aging in later life [14]. Emerging evidence has reported the association between long-term trajectory of obesity during adulthood and poorer brain health [15, 16]. Given the dynamic nature of weight status across the lifespan and reports of a potential late-life “protective” effect of obesity [17], a life-course perspective is therefore essential to capture how prolonged adiposity over several decades may contribute to metabolism-related brain decline. However, population-based evidence directly connecting life-course adiposity patterns to cerebral oxygen metabolism remains limited. Conceptually, novel approaches such as group-based trajectory modeling (GBTM) delineate decades-long adiposity patterns, enabling evaluation of their relevance to CMRO₂ in aging populations. Such cumulative exposure is expected to impact brain regions including the hypothalamus, thalamus, amygdala, and middle temporal gyrus, which are well-recognized for their roles in metabolic regulation [18–20]. Moreover, a previous study indicated that the hypothalamus, a key regulator of energy homeostasis, is associated with body mass [21], providing a potential mechanistic foundation for linking obesity trajectories, CMRO₂, and regional brain integrity.

Hence in this study, we aimed to examine the association between body shape, including global and abdominal indicators, and brain metabolism. We hypothesized that: (1) cross-sectionally higher late-life adiposity would be associated with lower CMRO₂; (2) CMRO₂ would partially mediate the relationship between obesity and neurodegeneration; 3) longitudinally, body shape trajectories from early adulthood until later life across four decades would be identified, and trajectories reflecting higher and/or more persistent adiposity would be associated with lower CMRO₂ and adverse regional alterations in metabolically relevant areas (e.g., the hypothalamus), reflecting a cumulative metabolic-load; 4) global cerebral metabolic status would relate to regional structural biomarkers that are sensitive to age-related brain metabolism.

## Methods

### Study population and study design

This study was part of the Chinese Study for the Prevention dementia among Community-Dwelling Older Adults (the Heritage Study [22]), a community-based cohort of older adults in Hangzhou, designed to investigate brain health and cognitive decline. From February 2023 to July 2024, 303 eligible community-dwelling older participants aged 50 and above were recruited from Gongshu District, Hangzhou, China. Participants underwent neuroimaging, physical examinations, and clinical investigations. This study was performed in accordance with the Declaration of Helsinki. Ethical approval was obtained from Zhejiang University.

Inclusion Criteria: Age > 50 years; Community-dwelling individuals; Capable of completing MRI and cognitive assessments; Provided written informed consent. Exclusion Criteria: Diagnosis of acute stage of stroke and transient ischemic symptoms; Diagnosis of Dementia; History of major psychiatric disorders (e.g., schizophrenia, major depressive disorder requiring hospitalization); Contraindications to MRI (e.g., pacemaker, metallic implants, severe claustrophobia); Poor image quality due to motion artifacts or technical issues; Evidence of significant brain pathology on MRI (e.g., large infarcts, tumors).

### Body shape measurements

Participants were weighed and measured while barefoot and wearing light clothing. Body Mass Index (BMI) was calculated as weight in kilograms divided by the square of height in meters (kg/m^2^) on the same day as the MRI scan. Additionally, self-reported height and weight data were retrospectively collected at multiple time points (at ages 25, 40, and 60 years) based on Stunkard’s Figure Rating Scale [23]. According to Chinese guidelines for healthy Chinese adults, BMI was categorized into four groups: underweight (< 18.5), normal weight (18.5 to 23.9), overweight (24.0 to 27.9), and obese (≥ 28.0) [24].

Standing waist circumference (WC) was measured at the level of the umbilicus using an ergonomic measuring tape and recorded to the nearest 0.1 cm. WC was treated as a continuous measure of abdominal obesity, reflecting central adiposity [13]. In later life, waist circumference tends to increase, making indices like the Body Roundness Index (BRI) [25] particularly relevant for capturing abdominal adiposity, including normal-weight obesity phenotypes [26, 27]. The BRI, calculated from waist circumference and height (equation below), ranges from 1 to 16 and serves as an indicator of body fat percentage and visceral adipose tissue, with prior applications in Chinese cohorts [28]. Several studies have applied BRI tertiles for risk stratification [29, 30]. In the present study, BRI was similarly categorized into tertiles based on its distribution within the sample, yielding cut-off points at the 33rd (BRI = 3.72) and 67th percentiles (BRI = 4.66), which defined the low, medium, and high BRI groups.

$$BRI=364.2-365.5\times \sqrt{1-(\frac{{(\frac{WC}{2\pi })}^{2}}{{(0.5\times Height)}^{2}})}$$

### MRI acquisition and analysis

Magnetic Resonance Imaging was acquired on a MAGNETOM Prisma 3T scanner using a 64-channel receiver head coil (Siemens Healthcare, Erlangen, Germany). Foam padding was placed around the participants’ heads to make them comfortable and minimize head motion. All participants underwent T1 weighted magnetization-prepared rapid gradient echo (T1-MPRAGE), phase-contrast (PC) and T2-Relaxation-Under-Spin-Tagging (TRUST) MRI sequences [31]. Detailed MRI protocols can be found in the Supplementary Materials.

T1 MPRAGE segmentation was performed using an automatic processing tool, MRI Cloud (https://www.MRICloud.org; Johns Hopkins University, Baltimore, MD) to acquire brain volume quantification [32], including total brain volume and regional volumes. The severity of medial temporal lobe atrophy (MTA) was also assessed and was graded on coronal sections using the Scheltens’ Scale (0 = normal, 1 = mild, 2 = mild to moderate, 3 = moderate, 4 = severe) [33] by a neuroradiologist F.W with 15 years of experience. An MTA score beyond 2 was labeled as having significant MTA.

Cerebral metabolic rate of oxygen was measured using the TRUST and PC sequences. TRUST assessed the pure venous T2 at the superior sagittal sinus, which can be converted to global venous oxygenation (Yv), using a pre-established calibration curve [34]. Global cerebral blood flow was measured using PC MRI by quantifying flow in the four major cerebral arteries: the left and right internal carotid arteries and the left and right vertebral arteries. Regions of interest (ROIs) were manually delineated on complex difference magnitude images to identify these arteries. The segmentation was performed by Y.Y., an MR specialist with four years of experience. Flow rates, expressed in milliliters per minute, were calculated by integrating velocity across the delineated ROIs. Subsequently, Cerebral Blood Flow (CBF) was calculated by dividing the total blood flow by the total brain volume. Based on Fick’s principle [35], when fully oxygenated arterial blood flows through the capillaries, the surrounding brain tissue extracts oxygen; thus, CMRO₂ can be calculated as:

$${CMRO}_{2}=CBF\times \left(Ya-Yv\right)\times Ca$$

Here, Ya (assumed to be 98% [36]) is the arterial oxygenation, and Yv is the venous oxygenation defined as the fraction of oxyhemoglobin in the venous blood. Ca is a constant representing the oxygen-carrying capacity of a unit volume of blood, and CMRO₂ was reported in μmol/100 g/min using Ca = 8.97 μmol O₂/mL blood based on the literature [8].

All MRI data were processed using in-house MATLAB scripts (version 2023a, MathWorks, Natick, MA).

### Statistical analysis

#### Descriptive comparisons

The characteristics of participants were examined using chi-square tests for categorical variables and ANOVA or Kruskal–Wallis test for continuous variables, as appropriate.

#### Covariates

Age, sex, and cardiometabolic conditions (hypertension, diabetes, hyperlipidemia, present/absent) were included as covariates in the models. Cardiometabolic covariates (hypertension, hyperlipidemia, diabetes) were determined from interviewer-administered medical histories and confirmation of physician diagnoses and current medications.

### Cross-sectional analyses

The association between body-shape and CMRO₂ was analyzed using a general linear model (GLM). For BMI models, the reference group was the normal-weight category (18.5 to 23.9 kg/m^2^); for BRI models, the reference was the lowest tertile. We applied the Johnson-Neyman procedure to identify the age range in which obesity was associated with significant hypometabolism.

#### Stratified analysis

Ordinal mediation analyses (R mediation package) were conducted overall and within the two age strata (< 70 and ≥ 70 years), treating MTA as an ordered categorical outcome. The 70-year cutoff was based on (1) prior literature suggesting hypoperfusion after age 70 [37], and (2) the cohort’s median age. The Jonckheere-Terpstra (JT) test was used to examine the monotonic association between CMRO₂ and MTA.

### Longitudinal analysis

#### Trajectory model

BMI trajectories were derived using group-based trajectory modeling, a specialized form of finite mixture modeling that assumes distinct latent subgroups with similar longitudinal BMI patterns. BMI values at the same four time points (ages 25, 40, 60, and current) were modeled using a censored normal distribution, and the optimal number of trajectory groups was determined based on the Bayesian Information Criterion (BIC), posterior probabilities, and group interpretability [38] (see eTable 1 in the Supplement).

#### Regional brain analyses

Eight metabolism-related ROIs were examined (left/right hypothalamus, thalamus, amygdala, and middle temporal gyrus) [39–41]. As a screening step, a Jonckheere-Terpstra test across BMI trajectory groups was applied; ROIs with a P for trend advanced to modeling. Additionally, GLMs were used to evaluate the associations between BMI trajectory groups and brain imaging markers (CMRO₂ and ROI volumes), adjusting for age, sex, hypertension, hyperlipidemia and diabetes. Finally, GLMs were used to assess the association between CMRO₂ and regional brain volumes, adjusting for age, sex, and total brain volume.

To account for multiple comparisons, false discovery rate correction using the Benjamini–Hochberg procedure was applied. This method is commonly used in neuroimaging studies because it provides effective control of multiple comparisons while preserving statistical power [42].

## Results

A total of 303 participants were included in the analyses. Sample characteristics across different body shape groups were shown in Table 1.

**Table 1:** Characteristics of participants

	Whole participants (*N* = 303)	Underweight/Normal (*N* = 153)	Overweight (*N* = 109)	Obese (*N* = 41)	*P* value
Age, Mean (SD)	67.9 ± 8.73	67.1 ± 8.64	68.4 ± 8.86	70.0 ± 8.49	0.122
Gender (Male)	94 (31.0%)	38 (24.8%)	41 (37.6%)	15 (36.6%)	0.062
Anthropometric indices					
Current BRI	4.30 ± 1.34	3.55 ± 0.92	4.65 ± 0.87	6.12 ± 1.52	** < 0.001**
Current BMI	24.1 ± 3.21	21.7 ± 1.81	25.5 ± 0.96	29.7 ± 1.54	** < 0.001**
Current WC	87.1 ± 10.1	81.2 ± 7.62	90.2 ± 6.87	100 ± 9.16	** < 0.001**
Current Height	160 ± 7.57	160 ± 7.67	160 ± 7.74	160 ± 6.88	0.881
Life styles					
Smoking history	63 (20.8%)	24 (15.7%)	30 (27.5%)	9 (22.0%)	0.065
Drinking history	97 (32.0%)	44 (28.8%)	42 (38.5%)	11 (26.8%)	0.194
Cardiovascular Disease					
Hypertension	141 (46.5%)	59 (38.6%)	55 (50.5%)	27 (65.9%)	**0.005**
Hyperlipidemia	106 (35.0%)	46 (30.1%)	38 (34.9%)	22 (53.7%)	**0.021**
Diabetes mellitus	61 (20.1%)	26 (17.0%)	24 (22.0%)	11 (26.8%)	0.326
Neuroimaging Markers					
CBF	58.2 ± 8.91	59.7 ± 8.78	56.8 ± 8.51	56.2 ± 9.61	**0.011**
CMRO_2_	206 ± 31.9	211 ± 32.7	202 ± 30.9	200 ± 29.6	**0.041**
MTA	1 [0, 3]	1 [0, 3]	1 [0, 3]	1 [0, 3]	0.137
Left Hypothalamus volume	528 ± 55.7	516 ± 54.7	542 ± 53.2	538 ± 57.3	0.101
Right Hypothalamus volume	592 ± 61.0	587 ± 59.2	595 ± 56.9	603 ± 76.3	0.260
Left Thalamus volume	4850 ± 462	4840 ± 406	4870 ± 530	4860 ± 470	0.831
Right Thalamus volume	4980 ± 474	4990 ± 428	4970 ± 518	4990 ± 527	0.962
Left Amygdala volume	1570 ± 198	1570 ± 183	1570 ± 219	1600 ± 195	0.618
Right Amygdala volume	1780 ± 212	1780 ± 190	1770 ± 236	1820 ± 227	0.453
Left MTG volume	17,300 ± 2000	17,100 ± 1860	17,300 ± 2140	17,700 ± 2110	0.231
Right MTG volume	15,700 ± 1760	15,500 ± 1810	15,900 ± 1720	16,000 ± 1640	0.084

*BRI* body roundness index, *BMI* body mass index, *WC* waist circumference, *CBF* cerebral blood flow, *CMRO₂* cerebral metabolic rate of oxygen, *MTA* medial temporal atrophy, *MTG* middle temporal gyrus. Volume is measured in mm^3^

### Association between the body shape and age-related cerebral metabolism

Relative to normal weight, overweight showed an additional −1.12 $\mu$ mol/100 g/min per year decline in CMRO₂ (95% CI = (−1.96, −0.28), *p* = 0.009; FDR-adjusted *p* < 0.05) and obesity showed an additional −1.40 $\upmu$ mol/100 g/min per year decline (95% CI = (−2.63, − 0.17), *p* = 0.027; FDR-adjusted *p* < 0.05) after adjusting for sex and cardiometabolic conditions; underweight did not differ.

Considering BRI tertile groups, a dose-responsive pattern emerged: compared with the lowest tertile, the high BRI group exhibited an additional −1.31 $\mu$ mol/100 g/min per year decline (95% CI = (− 2.36, − 0.27), *p* = 0.014; FDR-adjusted *p* < 0.05), whereas medium BRI was not significant (β = −0.73, 95% CI = (−1.77, 0.30), *p* = 0.167; Table 2). Johnson-Neyman analysis indicated that the interaction was significant among older adults; specifically, when age exceeded 72.56 years, higher BMI was significantly associated with lower CMRO₂. Similarly, when age exceeded 76.88 years, higher BRI was also significantly associated with reduced CMRO₂ (eFigure 1 in the Supplement). Both BMI and BRI were significantly associated with reduced cerebral blood flow (BMI: β = −0.38, *p* = 0.016; BRI: β = −0.86, *p* = 0.024, eFigure 2 in the Supplement). However, no significant associations were observed between obesity indices and oxygen extraction fraction (BMI: β = 0.07, *p* = 0.352; BRI: β = 0.35, *p* = 0.072, eFigure 3 in the Supplement).

**Table 2:** Interactions between Age and BMI category, BRI tertile with CMRO2

	Model 1	*P*-value	Model 2	*P*-value
Underweight*age	−0.37(−2.37, 1.64)	0.722	−0.14(−2.14, 1.85)	0.889
Normal weight ^a^ *age	**Ref**		**Ref**	
Overweight*age	**−1.09(−1.93, −0.24)**	**0.012**^c^	**−1.12(−1.96, −0.28)**	**0.009**^c^
Obese*age	**−1.52(−2.77, −0.29)**	**0.016**^c^	**−1.40(−2.63, −0.17)**	**0.027**c
Low BRI^b^ *age	**Ref**		**Ref**	
Medium BRI *age	−0.63(−1.66, 0.40)	0.234	−0.73(−1.77, 0.30)	0.167
High BRI*age	**−1.37(−2.41, −0.33)**	**0.010**^c^	**−1.31(−2.36, −0.27)**	**0.014**^c^

Note: Model 1 adjusted for sex, Model 2 additionally adjusted for cardiometabolic conditions (hypertension, hyperlipidemia, diabetes mellitus)

^a^The reference group was participants with normal BMI

^b^The reference group was participants with lowest BRI tertile. *CMRO₂* cerebral metabolic rate of oxygen, *BMI* body mass index, *BRI* body roundness index

^c^P(FDR) < 0.05, corrected for false discovery rate (FDR) using the Benjamini–Hochberg method

### Age-stratified association between the body shape and CMRO₂

The associations between adiposity measures and CMRO₂ stratified by age group were presented in eTable 2 in the Supplement. In the older group, both BMI and BRI showed strong inverse relationships with CMRO₂. Relative to normal BMI, those categorized as overweight (β = −18.15 $\mu$ mol/100 g/min, 95% CI = (−33.06, −3.24)) or obese (β = −28.79 $\mu$ mol/100 g/min, 95% CI = (−50.09, −7.48)) had significantly lower CMRO₂. Similarly, individuals in the highest BRI tertile had substantially reduced CMRO₂ compared to those in the lowest tertile (β = −18.24 $\mu$ mol/100 g/min, 95% CI = (−33.01, −3.46)).

### Mediation analysis

**Fig. 1:**
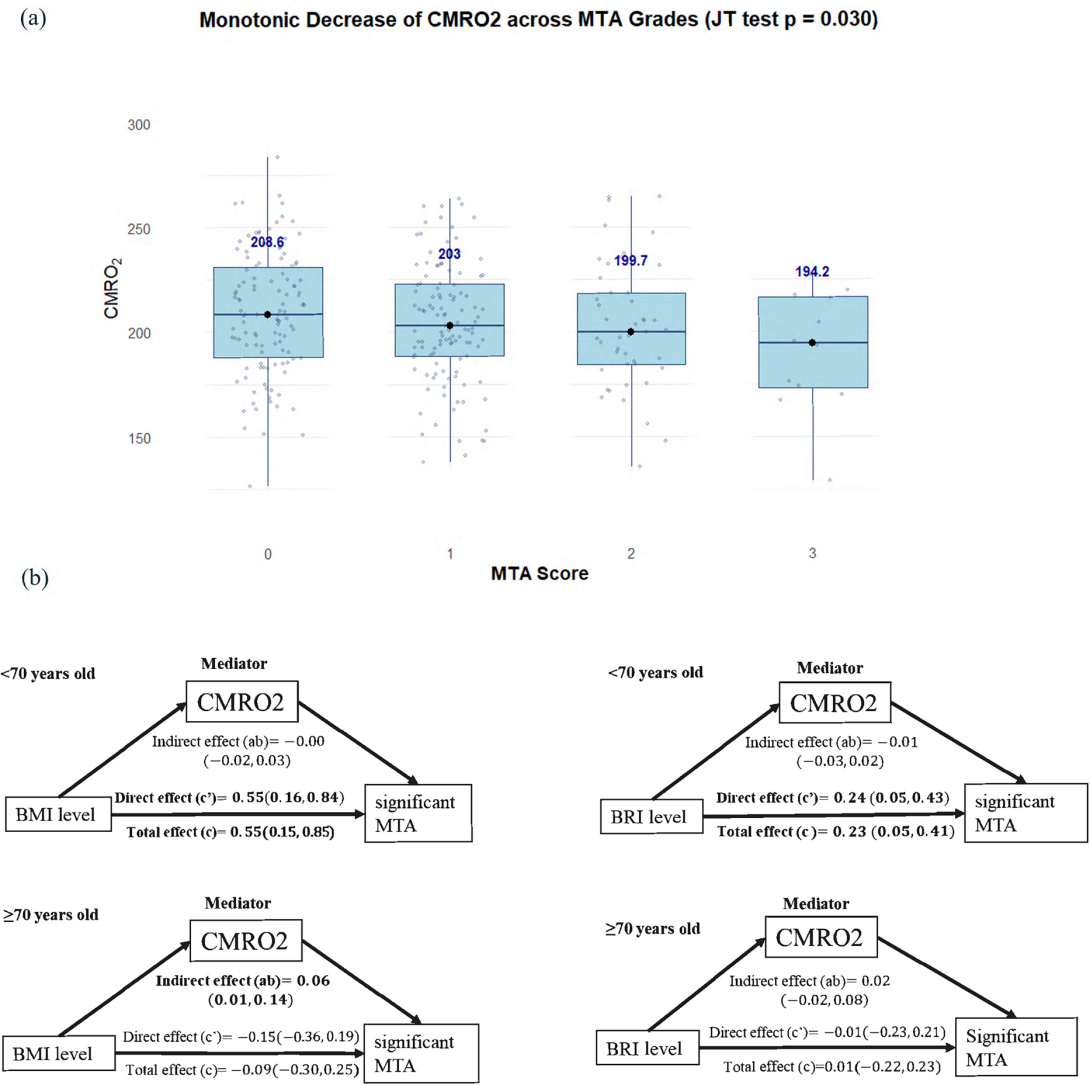
Decreased CMRO2 across MTA category grades and proposed mechanistic pathways. Note: **a** CMRO2 exhibited a decreasing trend with increasing MTA scores, as confirmed by the Jonckheere–Terpstra trend test (*P* = 0.030). Boxplots represent the interquartile range (IQR), with horizontal lines indicating medians. Individual data points are overlaid with jittered scatter. Median values for each MTA grade are additionally highlighted and annotated. The lowest median CMRO2 was observed in participants with the highest MTA score (Grade 3). **b** Each pathway reflects either a direct or indirect (mediated via CMRO2) effect of adiposity on MTA. This suggests that impaired brain metabolism may serve as a convergent pathway linking systemic adiposity and neurodegeneration especially in older adults

A significant monotonic decline in CMRO₂ was observed with increasing MTA grades, as indicated by the Jonckheere-Terpstra test (*P* = 0.030). Median CMRO₂ values steadily decreased from 208.56 $\mu$ mol/100 g/min in Grade 0 (n = 125) to 203.03 $\mu$ mol/100 g/min in Grade 1 (n = 121), 199.70 $\mu$ mol/100 g/min in Grade 2 (n = 45), 194.20 $\mu$ mol/100 g/min in Grade 3 (n = 12), suggesting an inverse association between MTA severity and cerebral oxygen metabolism (Fig. 1a).

Age-stratified mediation analyses suggested different pathways linking adiposity to MTA. In the < 70 years stratum, neither BMI nor BRI showed a significant indirect effect on MTA through CMRO₂. In the ≥ 70 years group (Fig. 1b), CMRO₂ partially mediated the relationship between BMI and MTA (Indirect effect by BMI: β = 0.06, 95% CI = (0.01, 0.14)).

### Association between the long-term body shape trajectories and cerebral metabolism

**Fig. 2:**
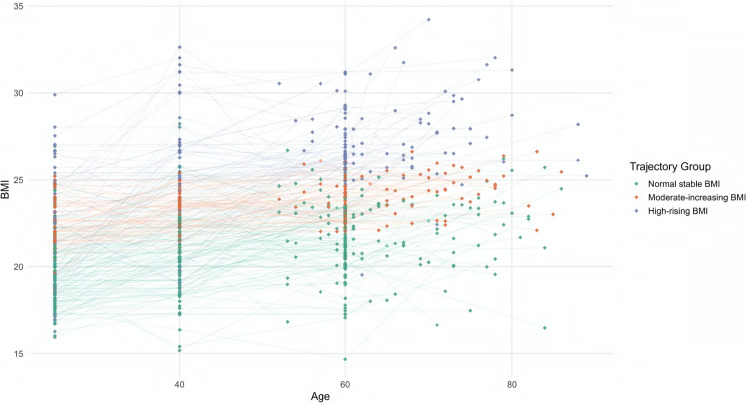
Trajectory of lifespan BMI. Note: For BMI, the trajectories were similarly categorized as: Group 1: "normal-stable" (consistently low BMI); Group 2: "moderate-increasing" (steady BMI increase); Group 3: "high-rising" (high initial BMI with a pronounced increase)

GBTM identified three BMI trajectories (normal-stable, moderate-increasing, high-rising, Fig. 2 and Supplementary eTable 3); mean age did not differ (*p* = 0.126). Anthropometrics (BRI, BMI, WC) rose monotonically across trajectories (all *p* < 0.01), with the high-rising group highest in value (BRI 5.32; BMI 27.7; WC 95.1 cm) and having more hypertension (60.6%), hyperlipidemia (45.5%), and diabetes (30.3%).

Compared to the normal-stable group, individuals in other trajectories exhibited significantly lower CMRO₂: moderate-increasing (β = −11.40, 95% CI = (−20.90, −1.90), *p* = 0.020; FDR-adjusted *p* < 0.05), high-rising (β = −12.23, 95% CI = (−23.56, −0.90), *p* = 0.036; FDR-adjusted *p* < 0.05). Regarding brain structure, the Jonckheere-Terpstra trend test demonstrated monotonic increases in left hypothalamic volume and bilateral middle temporal gyrus (MTG) volumes across BMI trajectory groups (eFigure 4). In GLMs, left MTG volume was higher in both moderate-increasing (β = 613.48, 95% CI = (20.20, 1206.75), *p* = 0.044; FDR-adjusted *p* < 0.05) and the high-rising group (β = 840.97, 95% CI = (137.65, 1544.30), *p* = 0.020; FDR-adjusted *p* < 0.05) as shown in Table 3. Right MTG volume showed a similar pattern (moderate-increasing: β = 615.70, 95% CI = (70.67, 1160.82), *p* = 0.028; FDR-adjusted *p* < 0.05; high-rising: β = 839.50, 95% CI = (193.35, 1485.72), *p* = 0.012; FDR-adjusted *p* < 0.05). Notably, left hypothalamic volume was significantly higher only in the high-rising group (β = 20.18, 95% CI = (0.57, 39.78), *p* = 0.045).

**Table 3:** Life-course adiposity trajectories and late-life brain metabolism and structure

	Trajectory group	Model 1	*P*-value	Model 2	*P*-value
CMRO2	normal-stable^a^	**Ref**		**Ref**	
	moderate-increasing	**−10.42(−19.99, −0.86)**	**0.034**	**−11.40(−20.90, −1.90)**	**0.020**^b^
	high-rising	**−10.65(−21.89, −0.59)**	**0.038**^b^	**−12.23(−23.56, −0.90)**	**0.036**^b^
Left Hypothalamus volume	normal-stable^a^	**Ref**		**Ref**	
	moderate-increasing	6.59(−9.37, 22.55)	0.334	8.51(−8.03, 25.04)	0.315
	high-rising	11.84(−3.65, 27.33)	0.093	**20.18(0.57, 39.78)**	**0.045**
Left MTG volume	normal-stable^a^	**Ref**		**Ref**	
	moderate-increasing	588.10 (−3.71, 1179.92)	0.052	**613.48(20.20, 1206.75)**	**0.044**^b^
	high-rising	**723.50 (31.25, 1415.69)**	**0.042**	**840.97(137.65, 1544.30)**	**0.020**^b^
Right MTG volume	normal-stable^a^	**Ref**		**Ref**	
	moderate-increasing	**593.9 (48.02, 1139.77)**	**0.034**^b^	**615.70(70.67, 1160.82)**	**0.028**^b^
	high-rising	**714.3 (75.84, 1352.82)**	**0.029**	**839.50(193.35, 1485.72)**	**0.012**^b^

Model 1 adjusted for the age gender and BRI; Model 2 additionally adjusted for cardiometabolic conditions (hypertension, hyperlipidemia, diabetes mellitus). *CMRO₂* cerebral metabolic rate of oxygen, *MTG* middle temporal gyrus

^a^The reference group was participants with normal-stable BMI group

^b^P(FDR) < 0.05, corrected for false discovery rate (FDR) using the Benjamini–Hochberg method

### Association between the CMRO₂ and metabolic-related regional volumes

**Fig. 3:**
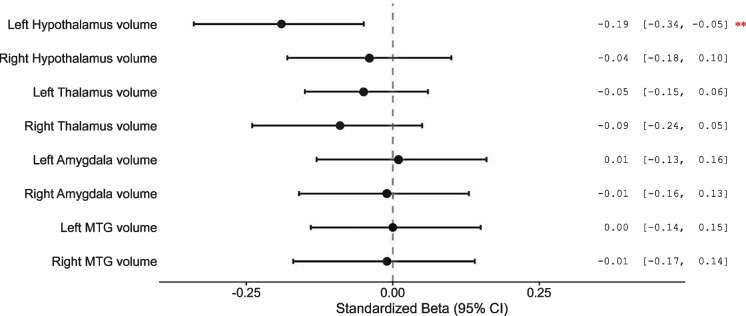
Associations between CMRO2 and metabolic-related brain region volumes. Note: This figure displays the standardized β coefficients and 95% confidence intervals from linear regression models assessing the association between cerebral metabolic rate of oxygen consumption (CMRO2) and regional brain volumes, adjusted for age, sex and total brain volume. CMRO2 showed a significant negative association with the left hypothalamus volume (β = −0.19, 95% CI: −0.34 to −0.05), while no significant associations were observed in other regions, including bilateral thalamus, middle temporal gyrus (MTG), and amygdala

We further examined the associations between CMRO₂ and regional brain volumes using standardized linear regression coefficients (Fig. 3). Among all regions assessed, only left hypothalamus volume was negatively associated with CMRO₂ (standardized β = −0.19, 95% CI = (−0.34, −0.05)), indicating that lower CMRO₂ was associated with higher left hypothalamus volume, the result suggests a region-specific relationship between cerebral metabolic function and brain morphology. In contrast, the associations with other regions, including the right hypothalamus, bilateral thalamus, bilateral amygdala, and bilateral middle temporal gyrus, were not statistically significant.

Exploratory mediation for BMI as exposure, left hypothalamus volume as mediator, CMRO₂ as outcome was carried out. However, the mediation effect was not statistically significant (total effect: β = 7.82, 95% CI (1.02, 14.63), direct effect: β = 7.14, 95% CI (0.33, 13.94), indirect effect: β = 0.68, 95% CI (−0.14, 2.10)).

## Discussion

In this community-based neuroimaging study of dementia-free older adults, we found that cross-sectionally, higher levels of BMI and BRI were associated with lower CMRO₂, suggesting that general and central obesity exacerbate age-related metabolic decline. Age-stratified analyses revealed that both general and central obesity in older adults were strongly related to reduced CMRO₂. Mediation analyses further indicated that CMRO₂ partially mediated the association between late-life adiposity and medial temporal lobe atrophy. Longitudinally, the long-term trajectory of higher BMI was associated with lower CMRO₂ and with larger left hypothalamus volume and bilateral MTG volume. Furthermore, lower CMRO₂ was associated with larger left hypothalamus volume, highlighting metabolism-related neuroimaging associations in normal aging.

### Obesity and cerebral metabolism in aging: Potential mechanistic pathways

In normal aging, metabolic downregulation has been proposed as an adaptive response to hemodynamic compromise, possibly arising under misery perfusion, lowering tissue oxygen demand to partially restore autoregulation and modestly reduce stroke risk, or alternatively reflecting selective ischemic neuronal loss without obvious infarction [43]. Given that both aging and adiposity affect cerebral metabolism, their effects may interact, suggesting that the impact of obesity on CMRO₂ could be age-dependent. In our study, both higher BMI and BRI were consistently associated with lower cerebral metabolism at older ages, especially above age 70.

CMRO₂ integrates CBF, oxygen extraction fraction (OEF), and arterial oxygen content, and provides a comprehensive indicator of the brain’s overall metabolic state. Prior literature, including work conducted in older adult populations, has shown that higher BMI is associated with reduced CBF, which may partially contribute to the lower CMRO₂ observed in individuals with obesity [10]. In contrast, the association between obesity and OEF appears to be age-dependent rather than uniformly decreasing [44]. Specifically, OEF tends to be elevated in middle-aged adults with obesity, a pattern that may reflect accelerated metabolic aging, whereas in adults older than 60 years, OEF begins to decline, potentially indicating reduced neuronal function. Taken together, these findings suggest that obesity-related reductions in CMRO₂ are at least partly driven by lower CBF, while OEF reflects distinct metabolic and neurophysiological mechanisms across age groups. This pattern aligns with our observations.

The underlying mechanisms between obesity and the brain could also be explained by insulin resistance, chronic low-grade inflammation, and cerebrovascular dysfunction [45]. Insulin supports vascular homeostasis through endothelial nitric oxide-mediated vasodilation; impaired insulin signaling can disturb vascular tone and promote cerebral hypoperfusion [46, 47]. Insulin resistance has also been implicated in cognitive impairment and neuroimaging abnormalities in type 2 diabetes mellitus and Alzheimer’s disease, and is associated with reduced cerebral glucose utilization and altered neuronal signaling that undermine synaptic efficiency [48, 49]. In parallel, obesity-related endothelial and glial perturbations and hypothalamic inflammation may disrupt energy homeostasis and compromise blood–brain barrier function [50]. Consistent with cerebrovascular dysfunction, higher adiposity has been linked to lower CBF in humans [10], while microvascular capillary transit-time heterogeneity can impair oxygen extraction and suppress CMRO₂ [51]. Together, these processes provide a mechanistic framework for interpreting our findings.

### Brain metabolism mediates the association between obesity and medial temporal atrophy

Hypoperfusion is an early pathological mechanism in neurodegeneration, likely driven by capillary constriction and other microvascular mechanisms [52]. In the predementia stages, FDG-PET work shows that hypometabolism emerges before measurable atrophy, supporting a sequence in which metabolic dysfunction precedes structural loss [53]. PET studies further demonstrate that medial temporal lobe CMRO₂ is distinctly reduced in mild-to-moderate dementia, and that this reduction is associated with memory performance [54]. Beyond disease stage, genetic susceptibility may modulate these links: APOE allele 4 carriers exhibit altered neurometabolic profiles [55]. In our healthy aging cohort, among adults ≥ 70 years, the association between adiposity and medial temporal lobe atrophy was mediated by impaired cerebral metabolism. Mechanistically, adiposity-related endothelial dysfunction, low-grade inflammation, and insulin resistance likely impair neurovascular coupling and oxygen delivery [56], resulting in lower CMRO₂ that partially mediates atrophy. This indirect pathway aligns with age-dependent vulnerability to metabolic stress and underscores the importance of managing adiposity and metabolic risks to preserve later-life brain metabolic health.

### The long-term effects of excess weight and its potential mechanisms

Long-term adiposity exposure, captured by life-course body-shape trajectories, was associated with greater regional volumetric differences, most notably in the enlargement of bilateral middle temporal gyri and the hypothalamus. This pattern is consistent with evidence, particularly in younger populations, linking higher adiposity to structural variation across metabolic-regulation hubs, including the thalamus, temporal lobe, hypothalamus [40, 57].

Large-sample MRI studies likewise show that higher BMI is associated with larger hypothalamic volume, and subregion-level analyses report enlargement of the superior anterior, inferior tubular, and posterior compartments, which are linked to appetite-regulating hormones [57, 58]. In our cohort of otherwise healthy older adults, we observed obesity-related volumetric enlargements of the hypothalamus and other regions, consistent with inflammation-related mechanisms. Converging evidence also points to altered hypothalamic microstructure with higher BMI, consistent with neuroinflammatory and gliotic processes [40]. Collectively, these observations are consistent with hypothalamic gliosis, neuroinflammation, and metabolic adaptation within circuits governing energy homeostasis and appetite regulation [59, 60].

Although the hypothalamus’s role in neurodegenerative disease differs from BMI-related variation [57], our aging cohort exhibited the same volumetric pattern, with higher BMI trajectories linked to larger hypothalamic volume. Notably, this pattern emerged only after sustained adiposity exposure, implying that detectable structural change requires prolonged metabolic burden.

Building on the link between obesity and global brain metabolism, we observed that lower CMRO₂ was associated with larger left hypothalamic volume, suggesting a coupling between metabolic impairment and structural variation in this key hub. Whether obesity directly alters hypothalamic volume, or whether the observed enlargement reflects inflammatory processes in obese individuals, remains unclear. The hypothalamus plays a central role in the homeostatic control of food intake and energy balance and serves as a key neural substrate linking peripheral adiposity to central metabolic processes [59]. In clinical neurodegenerative cohorts, such as amyotrophic lateral sclerosis (ALS), hypothalamic subregion volumes covary with metabolic, cognitive, and behavioral features [61]. Mediation analysis suggested a potential indirect effect of left hypothalamic volume in this relationship; however, the association did not reach statistical significance (p for trend = 0.08), likely due to limited sample size. Future studies, particularly experimental and longitudinal designs, are needed to clarify the causal direction between hypothalamic enlargement and reduced CMRO₂ in the context of obesity.

### Strengths and limitations

The strengths of this study include the use of brain metabolism MRI within a large community-based dataset. In addition, we leveraged life-course body-shape trajectories to capture long-term adiposity exposure, moving beyond reliance on single time-point BMI measures. Despite these strengths, several limitations should be acknowledged. First, phase contrast and TRUST MRI provide global CMRO₂ measurements; regional assessments in future studies may better capture region- or lesion-specific metabolism. Second, the relatively small sample size warrants interpretation of the associations between CMRO₂ and hypothalamic alterations. In addition, although trends were directionally consistent, not all between-group comparisons in the trajectory analyses remained significant after false discovery rate correction, underscoring the need for replication in larger samples. Nevertheless, reliance on self-reported BMI at earlier ages to construct weight trajectories may introduce recall bias, as participants may not accurately remember their height and weight during adolescence. Moreover, although we adjusted for several potential confounding factors, the absence of direct blood biomarkers (e.g., leptin) limits our ability to fully examine obesity-related pathology. Future large-scale longitudinal studies are needed to validate these findings.

## Conclusion

In this community-based sample, greater adiposity (BMI/BRI) was consistently associated with age-related reductions in CMRO₂. Among participants aged ≥ 70 years, mediation analyses supported a mediating role of CMRO₂ linking adiposity to medial temporal atrophy. Longitudinally, long-term exposure to higher BMI was associated with lower CMRO₂ and abnormal hypothalamic, middle temporal lobe volumes in later life; additionally, lower CMRO₂ was associated with larger left hypothalamic volume. Together, these findings provide insight into potential mechanisms linking obesity to brain aging and suggest that cumulative metabolic stress across the lifespan underscores the importance of proactive prevention and comprehensive risk-factor management to preserve brain metabolic health in later life.

## Supplementary information

Below is the link to the electronic supplementary material.

ESM 1 (DOCX 713 KB)

## Data Availability

The revised statement ensures that the datasets supporting the findings of this study are available upon reasonable request, with access evaluated by the corresponding author, Xin Xu, at Zhejiang University, in accordance with intellectual property protections, confidentiality agreements, and applicable ethical regulations.

## Declarations

### Data availability

The data supporting the findings of this study are available upon reasonable request. Requests to access the datasets will be reviewed by corresponding authors Xin Xu at Zhejiang University to evaluate compliance with intellectual property protections and confidentiality agreements, contingent upon adherence to applicable Chinese laws and ethical regulations.
