# Supplementary material for: Life-course body shape trajectories and cerebral oxygen metabolism in community-dwelling older adults
Source: GeroScience. 2026 Jan 12;48(1):183–96. doi: 10.1007/s11357-025-02082-x (PMC12972175; doi:10.1007/s11357-025-02082-x)
Supplement: Supplementary file 1 — (DOCX 713 KB) [file 11357_2025_2082_MOESM1_ESM.docx]

**Supplementary Materials**

**Article title:** Life-course Body Shape trajectories and Cerebral Oxygen Metabolism in Community-Dwelling older adults

**Submitted Journal:** GeroScience

**Author names:** Yifan Yan, MSc^1,2^, Yaping Zhang, MPH^1,2^, Xuhao Zhao, MPH^1,2^, Renwei Chen, MPH^1,2^, Shenghao Fang, BS^1,2^, Yi Zhou, BS^1,2^, Jingkai Huang, BS^1,2^, Fuyan Wang, MD^3^, Christopher Chen, FRCP^4^, Zixuan Lin, PhD^5^, Xin Xu, PhD^1,2,4^*

^1^School of Public Health, the Second Affiliated Hospital of School of Medicine, Zhejiang University, Hangzhou, Zhejiang, China

^2^ Key Laboratory of Intelligent Preventive Medicine of Zhejiang Province, Hangzhou, Zhejiang, China

^3^Department of Radiology, Sir Run Run Shaw Hospital, Zhejiang University School of Medicine, Hangzhou, Zhejiang, China

^4^Memory, Ageing, and Cognition Centre (MACC), Department of Pharmacology, Yong Loo Lin School of Medicine, National University of Singapore, Singapore, Singapore

^5^Key Laboratory for Biomedical Engineering of Ministry of Education, Department of Biomedical Engineering, College of Biomedical Engineering & Instrument Science, Zhejiang University, Hangzhou, Zhejiang, China

***Corresponding author**

Xin Xu, PhD

School of Public Health, the 2nd Affiliated Hospital of School of Medicine, Zhejiang University, China

Postal address: No. 866, Yuhangtang Road, Hangzhou, Zhejiang, P. R. China. 310058

Email address: xuxinsummer@zju.edu.cn;

**Supplementary materials catalogue**

**MRI protocols**

**Figure Legend**

**eFigure 1**. Interaction Effect of Age and BMI/BRI on Brain Metabolism

**eFigure 2.** Associations between obesity indices and cerebral blood flow

**eFigure 3.** Associations between obesity indices and oxygen extraction fraction

**eFigure 4.** Brain regional volumes across BMI trajectory groups.

**Table Legend**

**eTable 1-1.** Model fitting parameters of the BMI trajectory

**eTable 1-2**. Model fitting parameters of the BMI trajectory

**eTable 1-3**. Odds of Correct Classification of the Trajectory

**eTable 2.** Associations of BMI and BRI with CMRO2 in older adults (≥70 years)

**eTable 3.** Characteristics of each trajectory groups

**MRI protocols**

Parameters for T1 MPRAGE included the following: field-of-view (FOV)=256×256×1mm^3^, voxel size =1.0×1.0×1.0mm^3^, repetition time (TR) = 2.1s, inversion time (TI) =1.10s, echo time (TE) = 3.8ms, flip angle=12 degrees; T2 FLAIR sequence parameters were descripted as below: field-of-view (FOV)=220×220×$\times$5mm^3^, voxel size = 0.9×0.9×5.0mm^3^, repetition time (TR) = 7.5s, inversion time (TI) = 2.50s, echo time (TE) = 91.0ms, flip angle=150 degrees.

Sequence parameters of TRUST were: single slice, axial field-of-view (FOV)=220×220×5mm^3^, voxel size = 3.4×3.4×5.0mm^3^, repetition time (TR) = 3.0s, inversion time (TI) = 1.02s, echo time (TE) =3.9ms, labeling slab thickness = 100 mm, gap = 22.5 mm, four effective TEs (0.44, 40, 80, and 160ms) and total scan time = 1.2 minutes[1]**.**

Before the flow measurements, time-of-flight angiogram was performed to obtain the anatomical information of the feeding arteries of the brain. Imaging parameters of the angiogram were: TR=20 msec, TE=3.12 msec, flip angle = 18, FOV = 120mm, voxel size = 0.2*0.2*1.5 mm3, number of slices = 36, one 60-mm saturation slab positioned above the imaging slab, and scan duration = 46 seconds. To evaluate global CBF, Phase Contrast MRI (gradient echo) was performed targeting the four major cerebral arteries, i.e., the left and right carotid arteries (LICA and RICA) and the left and right vertebral arteries (LVA and RVA). PC MRI parameters included: TR = 8.6 msec, TE = 4.0 msec, flip angle = 20, FOV = 200×200×5 mm^3^, voxel size = 0.5×0.5×5mm^3^, Venc =40 cm/s, scan duration = 13 seconds[2].

Segmentation and negative-phase correction were performed by Y.Y., an MR specialist with four years of experience, in accordance with the phase-contrast MRI literature[2]. All MRI data were processed using in-house MATLAB (MathWorks, Natick, MA) scripts. Details of these MRI techniques and processing steps can be found in Lin et al[3,4].

**eTable 1-1.** Model fitting parameters of the BMI trajectory

| model | average posterior probability (APP) | | | |
| --- | --- | --- | --- | --- |
|  | G1 | G2 | G3 | G4 |
| traj_1 | 1 |  |  |  |
| traj_2 | 0.922561259 | 0.884378153 |  |  |
| traj_3 | 0.946431553 | 0.826633741 | 0.918532942 |  |
| traj_4 | 0.840744727 | 0.809720178 | 0.932532408 | 0.886954588 |

Note: Average posterior probability (APP) of group membership was tested to verify the model adequacy, APP beyond 0.70 is the criteria of good accuracy. The APP of group membership measured the likelihood for each participant to belong to its assigned group.

**eTable 1-2**. Model fitting parameters of the BMI trajectory

| model | Model fitting paramters | | | |  |
| --- | --- | --- | --- | --- | --- |
|  | aic | bic | caic | ssbic | hqic |
| traj_1 | 4354.417944 | 4368.730312 | 4371.730312 | 4359.203023 | 4359.89365 |
| traj_2 | 4185.178607 | 4218.574133 | 4225.574133 | 4196.343793 | 4197.955254 |
| traj_3 | 4105.605104 | 4158.083788 | 4169.083788 | 4123.150396 | 4125.682694 |
| traj_4 | 4080.329782 | 4151.891624 | 4166.891624 | 4104.25518 | 4107.708313 |

**eTable 1-3**. Odds of Correct Classification of the Trajectory

| model | G1 | G2 | G3 | G4 |
| --- | --- | --- | --- | --- |
| traj_3 | 20.20302 | 15.03278 | 27.24944 |  |
| traj_4 | 11.78474 | 21.52919 | 20.57503 | 55.55686 |

odds of correct classification (OCC) greater than 5.0 indicate that the model has high assignment accuracy, due to the small sample size, the three-trajectory group was chosen.

**eTable 2.** Associations of BMI and BRI with CMRO2 in older adults (≥70 years)

|  | Model 1 | Model 2 |
| --- | --- | --- |
| Underweight | -16.72(-54.26, 20.82) | -11.86(-49.18, 25.46) |
| Normal weight ^a^ | **Ref** | **Ref** |
| Overweight | **-17.38(-32.40, -2.35)** | **-18.15(-33.06, -3.24)** |
| Obese | **-31.69(-53.11, -10.28)** | **-28.79(-50.09, -7.48)** |
| Low BRI ^b^ | **Ref** | **Ref** |
| Medium BRI | 0.21(-15.57, 15.99) | -6.62(-22.82, 9.57) |
| High BRI | -11.73(-26.21, 2.75) | -**18.24(-33.01, -3.46)** |

**Note:** Age stratified analysis include participants age ≥70 years old. Model adjusted for sex, Model 2 additionally adjusted for cardiometabolic conditions (hypertension, hyperlipidemia, diabetes mellitus). (a) The reference group was participants with normal BMI; (b) The reference group was participants with lowest BRI tertile.

**eTable 3.** Characteristics of each trajectory groups

|  | Normal-stable group  (N=106) | moderate-increasing  (N=60) | high-rising  (N=66) | P value |
| --- | --- | --- | --- | --- |
| Age, Mean (SD) | 66.0 (9.04) | 68.8 (8.34) | 67.8 (8.19) | 0.126 |
| Gender (Male) | 24 (22.6%) | 20 (33.3%) | 25 (37.9%) | 0.081 |
| Current BRI | 3.64 (0.934) | 4.25 (0.753) | 5.32 (1.20) | **<0.01** |
| Current BMI | 22.0 (2.12) | 24.3 (1.22) | 27.7 (2.45) | **<0.01** |
| Current WC | 82.1 (7.80) | 86.9 (5.45) | 95.1 (7.84) | **<0.01** |
| Current Height | 160 (7.64) | 160 (6.61) | 161 (7.26) | 0.832 |
| Smoking history | 15 (14.2%) | 11 (18.3%) | 20 (30.3%) | **0.034** |
| Drinking history | 30 (28.3%) | 21 (35.0%) | 24 (36.4%) | 0.479 |
| Hypertension | 37 (34.9%) | 30 (50.0%) | 40 (60.6%) | **0.003** |
| Hyperlipidemia | 33 (31.1%) | 19 (31.7%) | 30 (45.5%) | 0.127 |
| Diabetes mellitus | 16 (15.1%) | 12 (20.0%) | 20 (30.3%) | 0.056 |
| GDS total score | 2.40 (2.81) | 2.00 (2.16) | 2.68 (2.59) | 0.280 |

**Note:** BRI: Body Roundness Index; BMI: Body Mass Index; WC: Waist Circumference; GDS: Geriatric Depression Scale.

**eFigure 1.** Interaction Effect of Age and BMI/BRI on Brain Metabolism


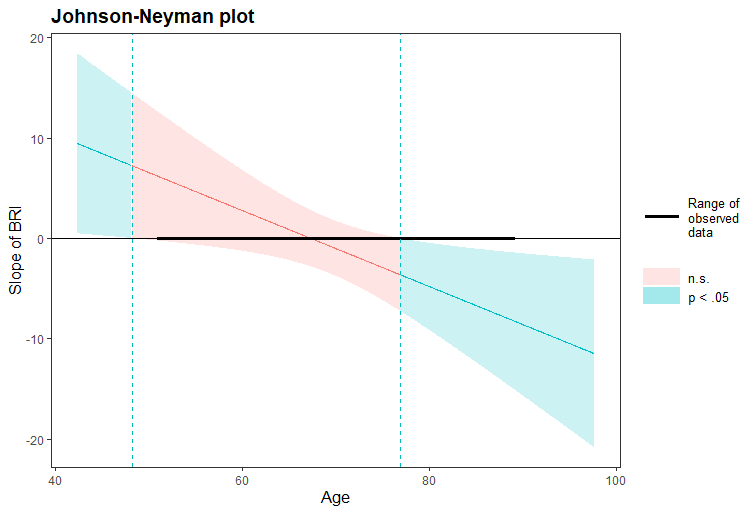


Age>76.88

(B) BRI and CMRO2


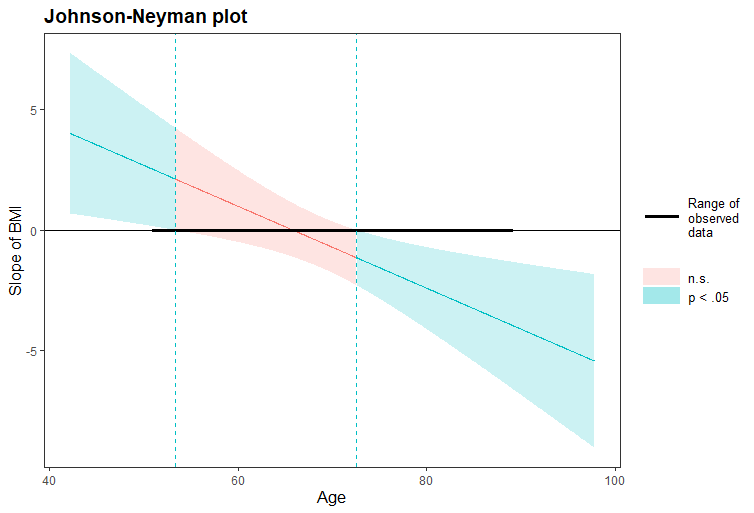


Age>72.56

1. BMI and CMRO2

**Note:** Interaction effects between age and adiposity indicators were detected.

(A) Among individuals older than 72.56 years, higher BMI predicted significantly lower CMRO2;

(B) A similar age-dependent association was observed for BRI, with a threshold at 76.88 years. All models were adjusted for sex.

**eFigure 2.** Associations between obesity indices and cerebral blood flow

β = -0.86, p=0.024*

β = -0.38, p=0.016*

**Note:** (A) Scatter plot showing the association between body mass index (BMI) and CBF with fitted linear regression line and 95% confidence interval. Higher BMI was associated with lower CBF (β = –0.38, *p* = 0.016).


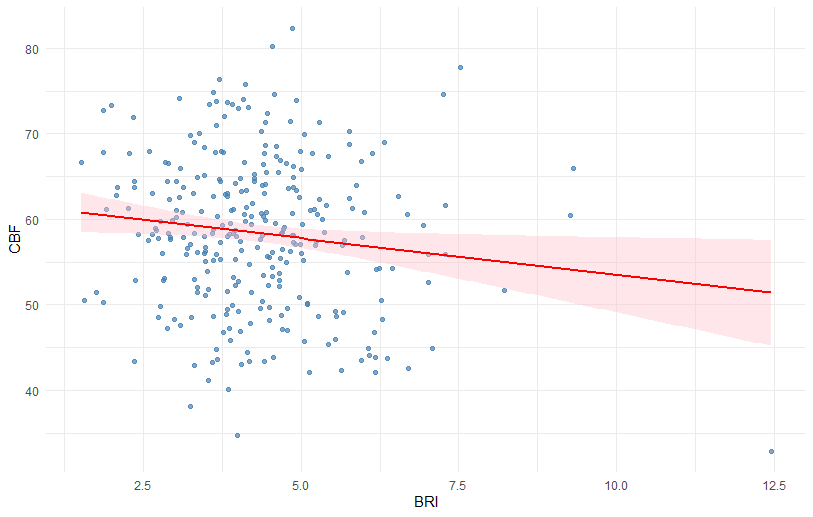

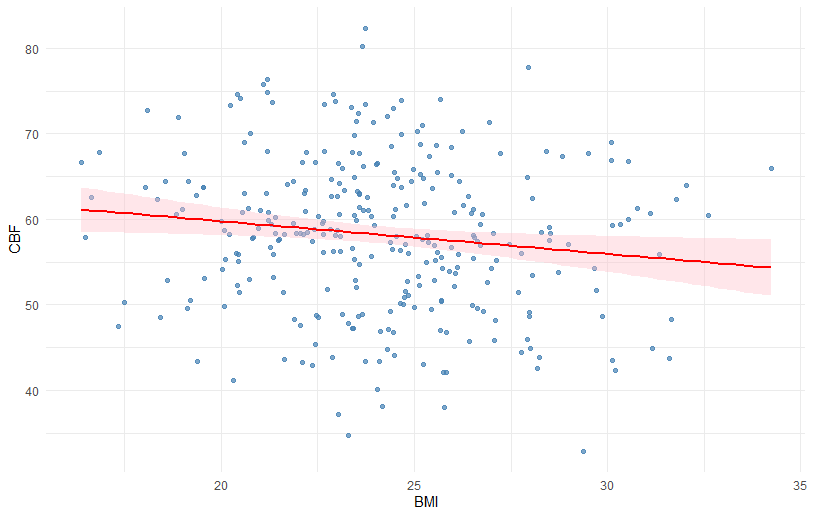


(A)

(B)

(B) Scatter plot showing the association between body roundness index (BRI) and CBF. Higher BRI was similarly associated with reduced CBF (β = –0.86, *p* = 0.024). Each dot represents one participant.

**eFigure 3.** Associations between obesity indices and oxygen extraction fraction

β = 0.354, p=0.072

β = 0.07, p=0.352


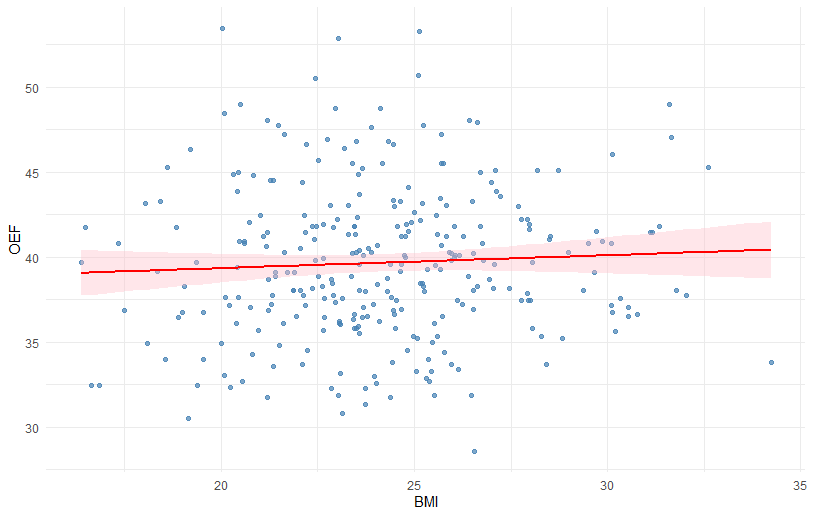

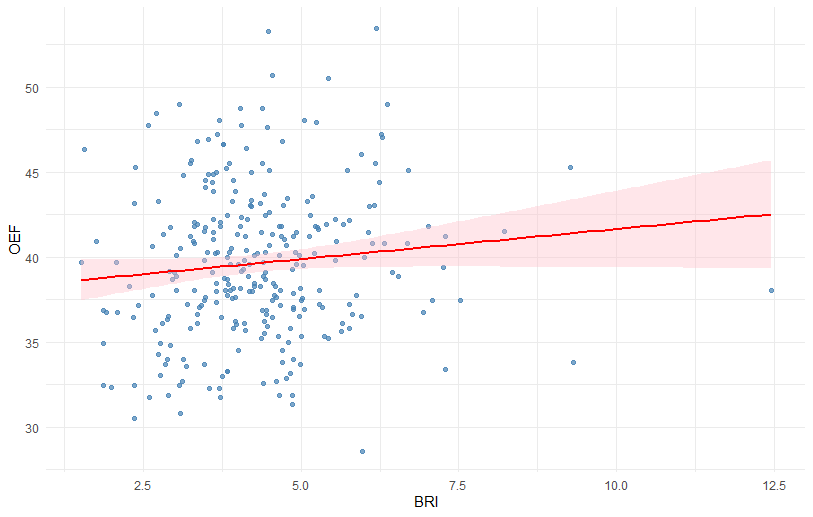


(A)

(B)

**Note:** (A) Scatter plot illustrating the association between body mass index (BMI) and OEF with fitted linear regression line and 95% confidence interval. BMI was not significantly associated with OEF (β = 0.07, p = 0.352).

(B) Scatter plot showing the association between body roundness index (BRI) and OEF. A positive trend was observed, although the association did not reach statistical significance (β = 0.354, p = 0.072). Each dot represents one participant.


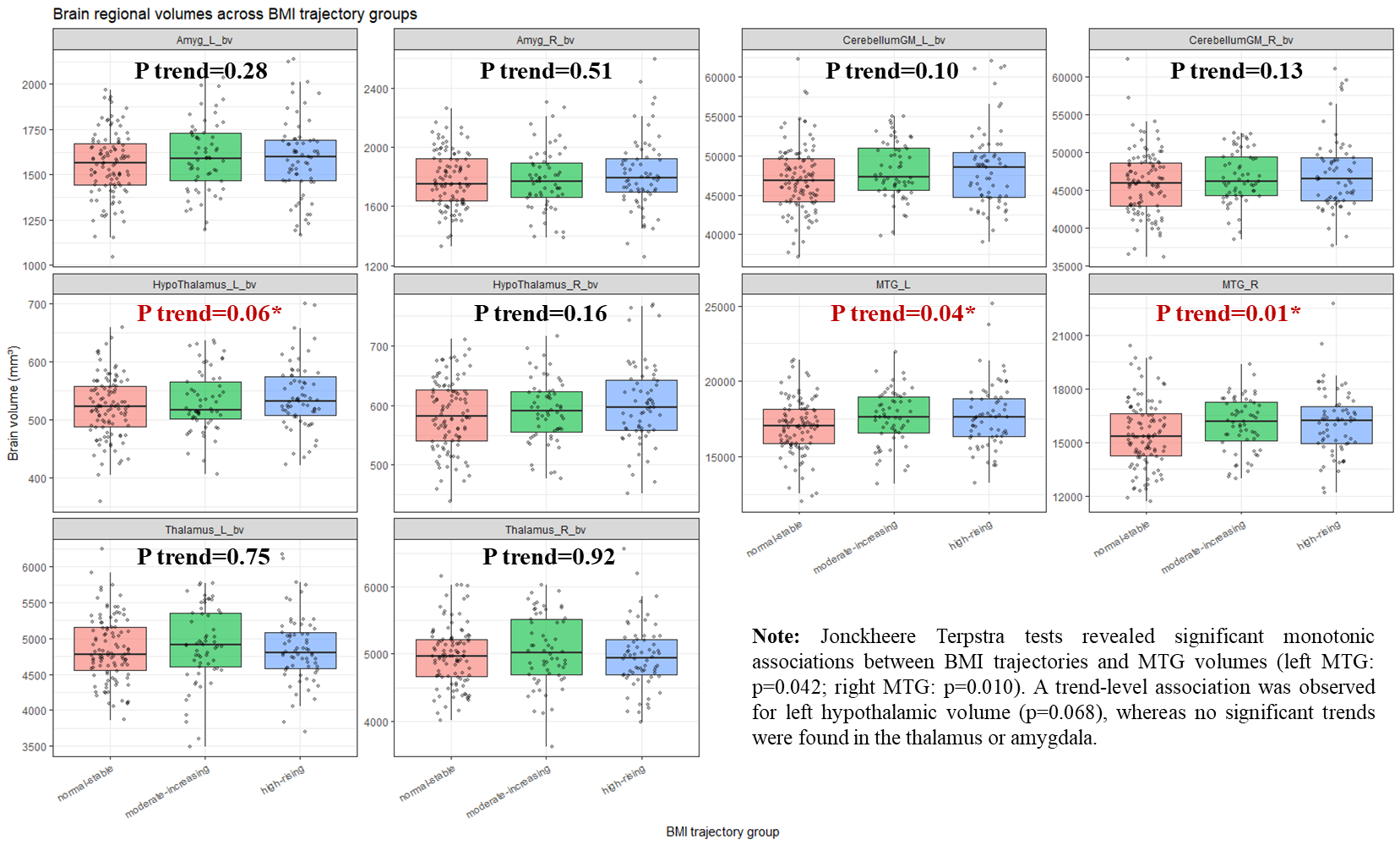
**eFigure 4.** Brain regional volumes across BMI trajectory groups.

**Refence:**

1. Lu H, Ge Y. Quantitative evaluation of oxygenation in venous vessels using T2‐relaxation‐under‐spin‐tagging MRI. Magn Reson Med. 2008;60:357–63. https://doi.org/10.1002/mrm.21627

2. Peng S, Su P, Wang F, Cao Y, Zhang R, Lu H, et al. Optimization of phase‐contrast MRI for the quantification of whole‐brain cerebral blood flow. J Magn Reson Imaging. 2015;42:1126–33. https://doi.org/10.1002/jmri.24866

3. Lin Z, Sur S, Soldan A, Pettigrew C, Miller M, Oishi K, et al. Brain Oxygen Extraction by Using MRI in Older Individuals: Relationship to Apolipoprotein E Genotype and Amyloid Burden. Radiology. 2019;292:140–8. https://doi.org/10.1148/radiol.2019182726

4. Lin Z, Lim C, Jiang D, Soldan A, Pettigrew C, Oishi K, et al. Longitudinal changes in brain oxygen extraction fraction (OEF) in older adults: Relationship to markers of vascular and Alzheimer’s pathology. Alzheimers Dement. 2022;alz.12727. https://doi.org/10.1002/alz.12727
